# Sensor‐Equipped Digital Technologies for Monitoring and Detecting Depressive Disorders: A Systematic Review

**DOI:** 10.1002/hsr2.71743

**Published:** 2026-03-02

**Authors:** Milad Rahimi, Kimia Abrishamifar, Shadi Hazhir, Hossein Valizadeh, Aynaz Nourani, Bahlol Rahimi

**Affiliations:** ^1^ Health and Biomedical Informatics Research Center Urmia University of Medical Sciences Urmia Iran; ^2^ Department of Medical Informatics, School of Allied Medical Sciences Urmia University of Medical Sciences Urmia Iran; ^3^ Department of Health Information Management, School of Management & Information Sciences Shiraz University of Medical Science Shiraz Iran; ^4^ Department of Health Information Technology and Management, School of Allied Medical Sciences Shahid Beheshti University of Medical Sciences Tehran Iran; ^5^ Department of Health Information Management, School of Health Management and Information Sciences Iran University of Medical Sciences Tehran Iran

**Keywords:** depression, mental health, sensor, smartphone, wearable

## Abstract

**Background and Aims:**

Depression is a common and chronic mental health problem, and the diagnosis and management of depression require continuous monitoring. In this review study, sensor‐based digital tools for the diagnosis and management of depression were examined. The effectiveness, usability, and limitations of these tools were evaluated and discussed.

**Methods:**

This systematic review was conducted in November 2025 using databases including IEEE, PubMed, Scopus, and Web of Science. The search was performed in accordance with PRISMA guidelines. Peer‐reviewed studies that had used digital technologies for the diagnosis, monitoring, or intervention in depression were identified. Eligible articles were included in the study after full‐text assessment.

**Results:**

In total, 41 studies met the inclusion criteria. Sample sizes in the studies ranged from 5 to 3936 participants. The study populations covered a wide range, from adolescents to older adults. Most investigations addressed various depressive disorders; some also referred to bipolar disorders or psychological distress. Overall, digital tools were categorized into smartphones, wearables, hybrid systems, and innovative platforms. These tools often used sensors such as global positioning systems (GPS), accelerometers, and heart rate monitors. Speech and facial analyzers were also employed. Data collection was carried out through active and passive monitoring of behavior, physiology, and mood.

**Conclusion:**

Sensor‐based digital tools have the capability to monitor and record the complex symptoms of depression. These data can also be used for personalized care. However, robust and standardized validation is required for clinical implementation. Future research should focus on long‐term engagement and scalability of these tools while maintaining confidentiality and sensitivity, with an emphasis on specific types of depression.

## 1 Introduction

Depression is a complex and multifaceted mental health condition that, according to the World Health Organization (WHO), affects approximately 280 million people worldwide [1]. This pervasive disorder does not distinguish between age, gender, and socioeconomic boundaries and manifests in diverse forms across different populations [2]. Depression arises from a combination of environmental, psychological, neurobiological, and genetic factors and is characterized by symptoms such as persistent sadness, fatigue, sleep disturbances, and changes in appetite [3]. Despite its remarkable prevalence and substantial consequences, factors such as social stigma, insufficient awareness, and limited access to care continue to impede timely diagnosis and effective intervention [4].

Traditional methods for diagnosing depression, such as self‐report questionnaires and clinical interviews, although essential, are often insufficient in capturing the subtle and dynamic nature of depression [5, 6]. These limitations underscore the need for more precise, continuous, and objective approaches to improve mental health assessments and interventions. Recent advances in digital health technologies, by providing innovative tools for real‐time monitoring of mood, behaviors, and physiological states, have demonstrated the potential to transform the way these gaps are addressed [7].

Digital technologies, including smartphones, wearable devices, and sensors, by leveraging their capacity to unobtrusively collect multidimensional data, have introduced new paradigms for monitoring depression [8]. These tools facilitate a more comprehensive understanding of the physical, psychological, and social dimensions of human behavior and generate deeper insights into mental health [9]. Passive sensing technologies, such as accelerometers, GPS, and biometric sensors, enable continuous and real‐time data collection and record vital indicators such as physical activity, sleep quality, heart rate, and social interactions [10, 11]. In contrast, active sensing approaches, which require user input (such as self‐reports or surveys), complement passive methods by providing contextual and self‐perceived data on emotional states [12, 13]. Together, these approaches integrate engineering innovation with clinical practice and create new opportunities for early diagnosis, personalized treatment, and preventive management of mental health [14, 15].

Recent developments in sensor‐based technologies highlight their capacity to bridge the gap between mental health assessment and timely intervention [16]. By combining advanced data analytics with continuous monitoring, these tools enable healthcare providers to make more informed decisions, enhance medication adherence in patients, and support access to psychotherapy or social resources [17, 18]. Despite this promising potential, the adoption of these tools in clinical practice raises important questions regarding their mechanisms, data reliability, and their impact on improving patient outcomes.

The aim of this systematic review is to provide a comprehensive evaluation of the current landscape of sensor‐based digital technologies for monitoring depression, with a focus on their mechanisms, data collection processes, and applications in the diagnosis, assessment, and management of depressive behaviors. By critically analyzing the capabilities and limitations of these tools, we seek to offer insights into their potential to transform mental health care and address the increasing global burden of depression.

## 2 Methods

This study was approved by the Ethics Committee of Urmia University of Medical Sciences (approval ID: IR.UMSU.REC.1403.214). This systematic review was conducted in accordance with the “Preferred Reporting Items for Systematic Reviews and Meta‐Analyses” (PRISMA) guidelines to ensure transparency, accuracy, and reproducibility.

### 2.1 Search Strategy

A comprehensive search strategy was developed to encompass three main domains: depression, behavior, and sensors and digital technologies. This search strategy used a combination of Medical Subject Headings (MeSH), keywords, and Boolean operators, which were tailored specifically for each database. Full details of the search strings for each database are provided in the Supplementary File.

The search was conducted in November 2025 in four major databases: PubMed, Scopus, IEEE Xplore, and Web of Science. Filters were applied to limit results to English‐language articles, review articles were excluded, and the publication period was restricted to studies published from 2019 onwards.

### 2.2 Study Selection

The study selection process followed the PRISMA guideline and is presented in Figure 2. In total, 4758 records were identified from the four databases. We then applied our predefined search limits, including publication within the past 6 years, English language, and original research articles, which led to the exclusion of 2184 records. A further 1002 duplicate citations were removed, leaving 1572 unique records for evaluation. At this stage, titles and abstracts were screened for consistency with our predefined inclusion and exclusion criteria.

Of these 1572 records, 1403 were excluded at the title and abstract level. The remaining 169 articles were retrieved for full‐text review. During this more detailed assessment, 128 studies were excluded for reasons such as inappropriate study design, insufficient outcome data, or lack of alignment with the target population or an overly strong focus on the intervention. Ultimately, 41 studies met all eligibility criteria and were included for discussion and interpretation.

### 2.3 Eligibility Criteria

Eligibility criteria were predefined to ensure the validity and reliability of the review. Original, peer‐reviewed research articles that examined the monitoring or tracking of behaviors, activities, or symptoms in individuals with depression were included. Studies were eligible if they used a digital tool, such as a smartphone, mobile application, wearable device, or standalone sensor platform, to record data related to depressive symptoms. We considered studies across the full spectrum of depression, including major depressive disorder (MDD), bipolar disorder (BD), and psychological distress or clinically depressive symptoms. All experimental designs, including randomized clinical trials, non‐randomized intervention studies, observational and cohort studies, feasibility and pilot studies, case–control studies, and cross‐sectional studies, were eligible. In line with our search strategy, only full‐text articles published in English within the 6‐year period were included.

We excluded records that did not focus on the monitoring or tracking of behaviors or activities in individuals with depression. Nonexperimental publications such as narrative reviews, systematic reviews, meta‐analyses, books or book chapters, posters or conference abstracts without full text, case reports, and single‐case studies were also excluded. In addition, non‐English publications and studies for which the full text was not available were removed.

### 2.4 Data Collection and Extraction

Data collection and extraction were conducted systematically and transparently by the first three authors using a predefined extraction form. The aforementioned authors recorded key bibliographic details (first author, year of publication, and country), main study characteristics (study design, demographic characteristics of participants, sample size, and age), study duration, and the digital tools or technologies used. Information related to analytical methods, outcome measures, and the type of depression or mental health status was also extracted.

All extracted information was systematically documented to facilitate critical appraisal of the included studies. Any discrepancies in data extraction were resolved through group consensus.

## 3 Results

**Figure 1:**
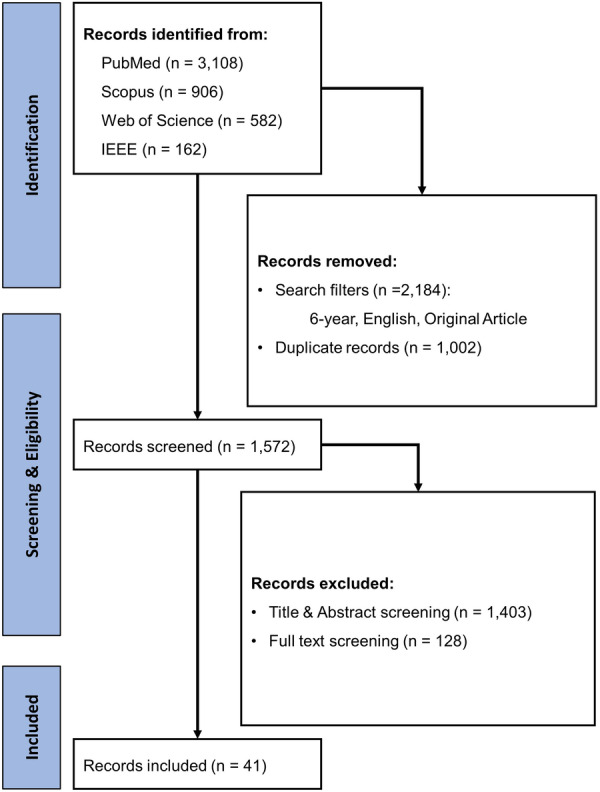
PRISMA flow diagram.

**Figure 2:**
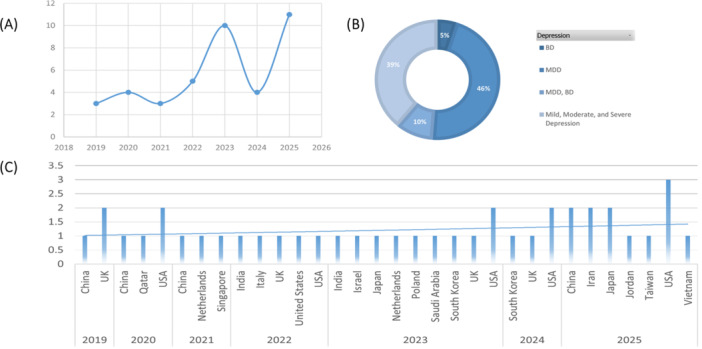
(A) Distribution of studies by publication year, (B) distribution by depression spectrum, and (C) study counts categorized by publication year and countries. Across the 41 studies, researchers used a variety of validated clinical instruments. Depressive symptoms were commonly assessed using scales such as PHQ‐9, PHQ‐8, PHQ‐4, BDI‐II, CES‐D, MADRS, HDRS, GDS‐30, IDS‐SR, and EPDS. Anxiety and psychological distress were recorded using measures including GAD‐7, STAI, BAI, DASS‐21, and the Kessler Psychological Distress Scale, while suicidality and associated risks were evaluated with tools such as the C‐SSRS and the Paykel Suicide Scale. Several studies incorporated broader constructs such as personality traits, self‐esteem, self‐efficacy, sleep quality, quality of life, and occupational and social functioning, using instruments such as PANAS, NEO‐FFI, RSE/RSES, WHO‐5, WHOQOL‐BREF, WSAS, and Neuro‐QoL, alongside structured diagnostic interviews and criteria (such as MINI, SCID‐5, and DSM‐5). Overall, this diverse set of measures highlights a multidimensional approach to understanding depression and mental health (Table 1).

The search strategy led to the identification of 41 eligible studies (Figure 1). Table 1 summarizes the key characteristics of these studies, and Figure 2 shows their distribution by year of publication (Figure 2A), spectrum of depression (Figure 2B), and country of implementation (Figure 2C). In total, 16 studies used interventional designs, including feasibility and pilot work [19, 20] and randomized clinical trials [21, 22, 23, 24, 25, 26, 27, 28, 29, 30, 31, 32, 33]. In addition, 17 studies employed observational‐analytical designs (cohort [39, 40, 41, 42, 43, 44, 45, 46], case–control [20, 36, 38], cross‐sectional [35, 47, 48], or longitudinal [49, 50, 51]). The remaining eight studies were descriptive [52, 53, 54] or methodological/technological development studies [55, 56, 57, 58, 59], which often focused on validating new assessment methods or platforms rather than testing specific clinical hypotheses. Study durations were heterogeneous, ranging from short, intensive monitoring periods of a few days to several weeks, to long‐term follow‐up designs of 1–2 years in large cohort studies.

**Table 1:** Summary of key characteristics and findings from included studies.

Ref	Study type	Number of participants	Age	Duration of study	Clinical measurements
[19]	Analytical Interventional (Feasibility)	24	18	1 month	The Global Physical Activity Questionnaire Kessler Psychological Distress Scale
[20]	Analytical Interventional (Pilot)	59		17 months	PHQ‐9 QIDS DSM‐5
[21]	Analytical Interventional (RCT)	34	13.8–15.4	5 weeks	CDRS‐R CDI C‐SSRS SCARED FACES‐IV IQ
[22]	Analytical Interventional (RCT)	57	> 60	9 months	PHQ‐9
[23]	Analytical Interventional (RCT)	72	≥ 60	8 weeks + 6 months follow‐up	GDS‐30 BAI PSQI WHOQOL‐BREF MoCA
[24]	Analytical Interventional (RCT)	73	64.5	1 month	CES‐D
[25]	Analytical Interventional (RCT)	73	Mean 40.4 ± 10.7	4‐week intervention + 2‐month follow‐up	PHQ‐4 CBTSS
[26]	Analytical Interventional (RCT)	84	21.6	2 weeks	CES‐D
[27]	Analytical Interventional (RCT)	91	≥ 20 (Mean 21.44 ± 0.96)	12 weeks	TDICS CMHC ATSPPH‐SF ESAS‐R
[28]	Analytical Interventional (RCT)	100	53.3	5 months	PHQ‐8 RSES ESQ
[29]	Analytical Interventional (RCT)	108	20–35	From ~30 weeks of gestation to 6 weeks postpartum	EPDS PHQ‐9 HPLP II
[30]	Analytical Interventional (RCT)	127	Mean 60.9 ± 9.9	~10–16 weeks (8‐week intervention)	PHQ‐9
[31]	Analytical Interventional (RCT)	345	59.33	6 months	BDI‐II WHO‐5 Paykel Suicide Scale (PSS) SEMCD6 Neuro‐QoL BIPQ‐Short Form SCS‐SF RAPA CIASS
[32]	Analytical Interventional (RCT)	708	15–25	12 months	MINI PHQ‐9 PHQ‐8 without sleep item ISI GAD‐7
[33]	Analytical Interventional (RCT)	3936	≥ 18	6‐week acute phase (+ up to 26–50 weeks)	PHQ‐9 GAD‐7 ISI SWEMWBS
[34]	Analytical Interventional (Single‐Arm Open Trial)	17	Mean 34.4 ± 3.1	10 weeks	PHQ‐9 GAD‐7 MINI
[35]	Analytical Observational (Cross‐Sectional)	25	18–48	4 weeks	PHQ‐9
[36]	Analytical Observational (Case‐Control)	40	18–69	14 days	HDRS CGI
[37]	Analytical Observational (Case‐Control)	55	20 – 69	Some days	MADRS
[38]	Analytical Observational (Case‐Control)	75	25.80	8 min and 33 s per patient	PHQ‐9 GAD‐7 MINI‐7 DSM‐5 Hamilton Depression Rating Scale
[39]	Analytical Observational (Cohort)	202/111/172 (across 3 cohorts)		10 weeks	PHQ‐4 BFI‐10 (OCEAN)
[40]	Analytical Observational (Cohort)	40	30–32	2 months	HDRS
[41]	Analytical Observational (Cohort)	45		2 months	MADRS PHQ‐8
[42]	Analytical Observational (Cohort)	66		7 months	PHQ‐9 GAD 7
[43]	Analytical Observational (Cohort)	182	18–25	17 months	PHQ‐9 QIDS
[44]	Analytical Observational (Cohort)	200	> 18	24 months	MEQ ASRS PHQ‐9 MADRS YMRS SCID‐5 DASI
[45]	Analytical Observational (Cohort)	600		Up to 2 years	IDS‐SR PHQ‐8 GAD‐7 WSAS BIPQ
[46]	Analytical Observational (Cohort)	766	48.61	7 days	PHQ‐9
[47]	Analytical Observational (Cross‐Sectional)	120	23.57	1 month	CES‐D
[48]	Analytical Observational (Cross‐Sectional)	534		2 years	PHQ‐4 PANAS BDI‐II PHQ‐4
[49]	Analytical Observational (Longitudinal Study)	16	22	1 month	PANAS STAI NEO‐FFI RSE BDI AQ
[50]	Analytical Observational (Longitudinal Study)	51	36.2	15 months	HDRS YMRS
[51]	Analytical Observational (Longitudinal Study)	142	55.1	Every 60 days for a year	DSM‐5
[52]	Descriptive (Cross‐Sectional)	40	25.5	2 weeks	MADRS
[53]	Descriptive (Cross‐Sectional)	59	35	12 months	PANAS DASS‐21 SIGHD‐IDSC
[54]	Descriptive (Cross‐Sectional)	290	33	14 days	PHQ‐9
[55]	Methodological and Technological Development Study	5	31		
[56]	Methodological and Technological Development Study	60	18– 6	1 month	BDI‐II SAI, TAI
[57]	Methodological Study	25			
[58]	Methodological Study	142	21.5	5 days during the winter	BDI‐II
[59]	Methodological Study	189			PHQ‐8

### 3.1 Demographic characteristics of participants

Across the 41 studies, on average, approximately 241 participants were enrolled per study. This mean was skewed upwards by a small number of large trials (up to 3936 participants), whereas the smallest study included only five individuals. The median sample size was 73, indicating that many investigations were conducted on a small to medium scale. The age profile of participants was similarly broad. Reported mean values or ranges indicated that the samples included adolescents, young adults, adults, and older adults into their late 60s and beyond.

The clinical and background profiles of participants were diverse. While most studies recruited individuals diagnosed with MDD, other studies included individuals with BD, perinatal depression, patients with cancer or on hemodialysis with comorbid depression, and individuals experiencing subclinical depressive symptoms or psychological distress in social or occupational settings. Several cohort studies combined depression with anxiety or other psychiatric or medical comorbidities, reflecting the clinical complexity of depression in real‐world settings.

#### 3.1.1 Overview of Sensor‐Based Digital Tools

##### 3.1.1.1 Smartphones for Depression Monitoring and Intervention

Smartphones were a core component for data collection and delivery of interventions in the studies reviewed. Many studies used passive sensing capabilities such as touchscreen interactions, app usage, call and SMS logs, GPS, accelerometer data, and Wi‐Fi connections to capture fine‐grained behavioral footprints related to depressive symptoms. For example, one study [47] used Android devices to record touchscreen behavior, call and SMS activity, and social media use in order to identify social interaction patterns associated with depression. Other studies combined the number of app uses, typing intervals, duration of use, location entropy, and sleep disruption indices across both Android and iOS operating systems [51]. Another study monitored physical movement and communication patterns to derive digital biomarkers for diagnosis and treatment response in adolescents with MDD [21].

In populations with BD, smartphone‐based monitoring was used to assess frequency and duration of calls, incoming and missed calls, characteristics of text messages, and self‐reported sleep and mood to identify the role and association of everyday communication behavior with the severity of depressive and manic symptoms [50]. Other work focused specifically on social interaction signals from SMS and call logs [20], or on passive sensing app and Wi‐Fi data [43], and GPS/accelerometer features combined with self‐reported PHQ‐8 scores to model depression severity across different diagnostic groups [41]. Beyond mood itself, some studies targeted physical activity and distress in working populations using self‐reported and digitally recorded activity in a deep‐learning‐based mobile application [19]. Further studies used remotely recorded speech features [36] or facial elements and video‐based features collected via smartphones to classify depressive states and predict depression scale scores [35, 57].

A substantial subset of studies also used smartphones not only for monitoring but as platforms for delivering psychological and behavioral interventions aimed at prevention or treatment. Several trials employed mobile applications on Android and iOS to deliver cognitive behavioral therapy (CBT) content, sleep diaries, mood check‐ins, and digital worksheets, while simultaneously logging app use and engagement over weeks to months. These interventions targeted a wide range of populations, including at‐risk young people with insomnia [32], adults with subthreshold depression [33], college students with depressive symptoms [27], pregnant women for prevention of postpartum depression [29], patients with heart failure in whom step counts and activity goals were used to reduce sedentary time and depressive symptoms [30], and older adults with late‐life depression receiving digital CBT [23]. Other work delivered behavioral activation via a gamification‐theory‐based app in perinatal populations [34] (Table 2).

**Table 2:** Smartphone sensor‐based digital tool characteristics.

Ref	Operating system	Data type	Primary function
[47]	Android	Metadata from smartphone usage: touchscreen interactions, call logs, SMS activity, and social app usage (e.g., WeChat and Sina Weibo)	Analyze social behavior on smartphones to identify correlations with depression, facilitating early diagnosis and improved social interaction for depressed individuals.
[51]	Android, iOS	Passively sensed behavioral data: app usage count, typing time interval, session duration, location entropy, and sleep disruption	Explore the multidimensional associations between depressive symptom severity and smartphone interaction behaviors, emphasizing within‐ and between‐person effects in longitudinal analysis
[21]	Android	Passively collected smartphone data: usage time, physical movement distance, number of phone calls and text messages, and call duration	Evaluate digital biomarkers for diagnosing depression and predicting treatment response in adolescents with major depressive disorder (MDD) using deep learning techniques
[50]	Android	Smartphone behavioral data: phone call frequency and duration, incoming and missed calls, text message frequency and length, and self‐reported sleep and mood assessments	Assess the relationship between smartphone‐collected behavioral data and depressive and manic symptoms in patients with bipolar disorder, enabling early detection and symptom severity
[20]	Android	Social interaction data: SMS logs and phone call logs, including outgoing and incoming messages and calls	Predict depression by analyzing social interaction patterns using machine learning models, focusing on distinguishing behaviors in outgoing SMS messages and phone calls
[43]	Android, iOS	Smartphone data: passively collected app usage data and institution WiFi infrastructure data	Predict behavioral and cognitive symptoms of depression using machine learning models based on passive smartphone and WiFi data, enabling objective and continuous symptom assessment.
[41]	Android, iOS	Smartphone data: GPS and accelerometer data, along with self‐reported PHQ‐8 measures	Evaluate passive smartphone measures as proxies for depression severity and compare their predictive performance with self‐report measures across multiple psychiatric diagnoses.
[19]	Android, iOS	Physical activity data (self‐reported and digitally recorded) and psychological distress scores (self‐reported via 6‐item Kessler Psychological Distress Scale).	To promote physical activity and reduce psychological distress (depression and anxiety) in the working population through passive monitoring and intervention, using a deep learning model within the ASHARE app
[36]	Not mentioned	Behavioral and emotional characteristics derived algorithmically from speech	To classify and monitor speech features related to MDD using a mobile app for remote speech recording and cloud‐based processing
[32]	Android, iOS	Self‐reported sleep diaries, questionnaires, and app logs (session completion)	Treatment of insomnia to prevent onset of major depressive disorder in at‐risk youth
[33]	Android, iOS	Self‐reported data, CBT worksheets for each skill, and app usage logs (time per chapter and chapters finished)	To reduce depressive symptoms in adults with subthreshold depression and, in the broader trial program, prevent future major depressive episodes
[34]	Not mentioned	Self‐report and app‐usage data, and app engagement metrics (days using app and number of activities completed).	Deliver behavioral activation via a gamified smartphone app to reduce perinatal depression and anxiety symptoms
[35]	Android	Facial behavior primitives (AUs, landmarks, head pose, etc.)	To collect facial behavior primitives in the wild and build machine‐learning models to detect depressive episodes and predict PHQ‐9 scores
[27]	Android, iOS	Self‐reported app data include daily mood and behavior entries, mood diary content, goals and progress, and engagement with stress‐relief and psychoeducational content, as well as questionnaire data on depressive symptoms, suicidal ideation, help‐seeking attitudes, and emotional self‐awareness at each time point	Treatment/self‐management of depressive symptoms in college students using CBT‐based strategies (cognitive reframing, self‐management, emotional/behavioral regulation)
[57]	Not mentioned	Facial video features (AUs, landmarks, and geometrical features)	Build models to recognize naturalistic depression from facial behavior in smartphone video (classification of depressive vs. non‐depressive)
[29]	Android, iOS	Educational materials (PDFs and audio files), self‐report questionnaire data, and demographic and fertility info	To promote health‐promoting behaviors in pregnancy and thereby prevent postpartum depression
[30]	Android, iOS	Automatically collected daily step counts (per day over weeks) plus goal‐tracking notifications from Samsung Health; self‐reported data: demographics, HF symptom burden (MSAS‐HF), comorbidity index, BMI, and depressive symptoms (PHQ)	Reduce sedentary time and increase light‐ or higher‐intensity physical activity in HF patients to improve symptoms and reduce depressive symptoms
[23]	Not mentioned	Self‐report scales and app usage logs	Reduce depressive symptoms and improve anxiety, sleep, and quality of life in older adults with LLD via digital CBT.
[25]	Android, iOS	Mood check‐ins, journal entries, CBT worksheets, and usage logs	Reduce anxiety and depressive symptoms and enhance CBT skills (especially self‐monitoring) among at‐risk employees via self‐guided CBT content

##### 3.1.1.2 Wearable Devices and Their Applications

In the reviewed studies, wearable devices placed on the wrist, chest, face, or finger were used to record physiological and behavioral data related to depression and mood disorders. Wrist‐worn devices were the most commonly used. Actigraphy‐based tools such as Actiwatch [37], ActiCal [40], and Actigraph GT3X+ [46] relied mainly on accelerometer‐based sensing to quantify sleep–wake patterns, activity, and circadian rhythm metrics. These were then used, via machine learning and deep learning techniques, to estimate symptom severity, treatment response, and diagnosis of depressive disorder. In one study, a multi‐sensor wearable platform [56] combined accelerometers, angular sensors, microphones, and temperature and humidity sensors to extract behavioral and speech‐based indices of mental state. Another study used the Fitbit Charge 2 wristband [54] to derive digital biomarkers such as step counts, heart rate, energy expenditure, sleep, and circadian rhythm for assessing depression risk. An Internet‐of‐Things (IoT)‐based social sensing system [49] integrated audio features with movement and environmental data to examine how physical and social environments relate to mental health and demonstrated how multimodal sensing can move beyond simple symptom tracking toward contextual understanding.

In addition to wrist devices, several studies used innovative wearables on other body sites to capture complementary aspects of emotional and physiological functioning. A multimodal chest‐worn device [55] combined electrocardiogram (ECG), galvanic skin response, temperature, and motion sensing to assess mental health and mood disorders during daily activities through intermittent multimodal snapshots. Face‐based sensing was implemented using Emteq's OCOsense™ smart glasses [38], which applied optomyography to measure facial muscle activity in response to emotional stimuli alongside subjective affect ratings, enabling detection of hyporeactivity of facial expressions in depression for remote monitoring and clinical assessment. The Oura ring [44], worn on the finger, integrated an accelerometer, gyroscope, and optical sensing to extract heart rate variability, sleep, and activity metrics and, via passive sensing, helped to predict illness episodes in BD. Lower‐limb motion sensors embedded in an “exergame” system during dialysis with virtual supervision [24] were used to deliver low‐intensity gamified exercise for hemodialysis patients with the explicit aim of reducing depressive symptoms (Table 3).

**Table 3:** Wearable sensor‐based digital tools' characteristics.

Refs	Body area	Data type	Sensor type	Tool name	Primary function
[55]	Chest	Electrocardiogram (ECG), galvanic skin response (GSR), temperature, bio‐motion	Wearable multimodal device with ECG, GSR, temperature and motion sensors	Low‐powered, flexible device (*)	Assess mental health and mood disorders during daily activities via noncontinuous, multimodal sensing
[38]	Face	Facial muscle activity during emotional stimuli, subjective emotional ratings	Glasses‐based optomyography sensor	Emteq's OCOsense™ smart glasses	Detect facial expression hyporeactivity in depression for remote monitoring and clinical assessment
[44]	Finger	Heart rate variability, sleep data, activity data	Accelerometer, gyroscope, infrared optical pulse sensor	Oura ring (*)	Forecast illness episodes in bipolar disorder using passive sensing, nonlinear techniques, and deep anomaly detection
[24]	Lower extremities	Motion data	Wearable motion sensors	Virtually supervised intradialytic exergame system (*)	Reduce depression symptoms in hemodialysis patients through gamified, low‐intensity, intradialytic exercise
[37]	Wrist	Human sleep and activity patterns	Accelerometer	Actiwatch	Monitor motor activity for mental health and depressive symptoms analysis
[40]	Wrist	Actigraphy data, circadian rhythm metrics, depression severity scores	Actigraphy sensor	ActiCal	Monitor circadian rhythm disruption and its association with antidepressant response in MDD patients
[46]	Wrist	Actigraphy data (minute‐level), sleep and movement patterns, PHQ‐9 scores	Actigraphy sensor	Actigraph GT3X+	Detect major depressive disorder (MDD) using sleep/movement data with ML and deep learning techniques
[56]	Wrist	Speech data, behavioral signals	Accelerometer, angular sensor, microphone, temperature and humidity sensor	Wearable sensor	Assess mental state objectively using speech, behavior, and deep learning techniques for improved psychological evaluation
[54]	Wrist	Steps, heart rate, energy expenditure, sleep patterns, circadian rhythm metrics	—	Fitbit Charge 2 (*)	Assess depression risk using digital biomarkers for physical activity, sleep, and circadian rhythm through machine learning
[49]	Wrist	Audio features (energy, entropy, brightness, formants), motion data, environmental data (temperature, humidity, light)	Microphone, accelerometer, gyroscope, temperature and humidity sensor	IoT‐based wearable social sensing platform (*)	Evaluate relationships between physical and mental health using speech, behavior, and environment sensing with feature fusion

##### 3.1.1.3 Hybrid Systems Integrating Multiple Technologies

Several studies implemented hybrid digital systems that integrated smartphone applications with wearable sensors, enabling remote and multiparametric measurement of depressive symptoms and related behaviors. In one study, a multiparametric remote measurement system [28] combined a smartphone application with a Fitbit device: the app delivered weekly PHQ‐8 assessments, visual progress feedback, notifications, and direct contact information for the research team, while the wearable continuously recorded passive activity and heart rate data. Similar architectures with active and passive data collection were used during psychotherapy for depression [42], as well as in long‐term cohorts in which smartphone apps logged location, screen status, physical activity, and sleep patterns, and wearables logged activity and sleep behaviors [48]. Other remote measurement technology (RMT) platforms extended this approach by explicitly distinguishing passive data streams from active smartphone‐based questionnaires on depressive symptoms and self‐esteem, complemented by continuous accelerometry and heart rate from a Fitbit Charge 2 [45]. The GLOBEM mobile sensing platform [39] was another example in this category, combining call logs, screen time, and location with sleep data and step counts derived from Fitbit devices to predict daily depression scores using sensor‐aware preprocessing and regression modeling.

Hybrid systems were also deployed as structured interventions that combined behavioral monitoring with targeted support. One trial [26] used a smartphone application and a Fitbit Flex wristband to deliver emotional and informational support aimed at reducing depressive symptoms and increasing physical activity, comparing an emotional‐plus‐informational support group with an information‐only group and a no‐intervention control group. Another study targeting bereaved older adults who had lost a spouse paired a smartphone application with an ActiGraph GT9X watch to monitor sleep, meals, and physical activity and compared digital monitoring alone with digital monitoring plus motivational health coaching and with usual‐care controls [22]. An e‐health system integrated a physiological smart shirt with a mobile application that delivered behavioral recommendations, mindfulness content, and CBT‐based strategies, thereby combining passive physiological monitoring with active psychological input to reduce depressive symptoms in individuals with severe somatic conditions [31].

One study [53] used the CHDR MORE™ smartphone application alongside wearables to record daily activity and self‐reported DASS‐21 and PANAS scores to quantify depression severity. Another study [58] focused on wearable sensors that recorded movement, skin conductance, heart rate, and blood volume pulse; these signals were transformed into image‐like representations using continuous wavelet transform and short‐time Fourier transform and were modeled with machine learning algorithms to predict depression severity and affective states. A further study developed a comprehensive diagnostic framework [52] that integrated a battery of neuropsychological tests, eye tracking, EEG‐based brain activity to quantify emotional perception biases, and passive behavioral monitoring of social interactions and mobility (Table 4).

**Table 4:** Hybrid sensor‐based digital tools characteristics.

Refs	Type of tool	Operating system/body area	Function	Data type
[28]	Multiparametric Remote Measurement Technologies	Smartphone app (Android/iOS) and wearable device (Fitbit)	Symptom tracking and engagement monitoring for major depressive disorder (MDD)	**1: Smartphone app** Weekly symptom assessments (PHQ‐8)
				Visual progress tracking and notifications
				Access to research team contact information
				**2: Wearable device**
				Continuous passive tracking (activity, heart rate, etc.)
				**3: In‐app components**
				Credible information, notifications, visual feedback
[26]	Smartphone‐based and Fitbit Flex devices	Smartphone and Fitbit Flex wristband targeting psychological and physical health	Emotional and informational support for reducing depressive symptoms and promoting physical activity	**1: Emotional and informational support group (Experimental Group 1)**
				Provided emotional support via mobile technology
				Delivered informational content related to mental health and physical activity
				**2: Informational support group (Experimental Group 2)**
				Delivered only informational content
				**3: Control group**
				No specific mobile technology intervention
[22]	Smartphone‐based app and ActiGraph GT9X watch	Digital tools for tracking sleep, meals, and physical activity, targeting older spousally bereaved adults	Behavioral intervention to monitor health metrics and provide coaching to reduce depression symptom burden	**1: Digital monitoring group**
				Tools for tracking sleep, meals, and physical activity
				**2: Digital monitoring + health coaching group**
				Same monitoring tools, plus motivational health coaching
				**3: Enhanced usual care group**
				Standard care with no specific digital intervention
[53]	Smartphone‐based application and wearable devices	Smartphone app (CHDR MORE™) for daily activity tracking	Remote monitoring of physiological, physical, and social activities to assess correlations with clinical depression severity scores	Self‐reported assessments (DASS‐21, PANAS)
				Smartphone‐based activity metrics (e.g., steps‐per‐minute, travel duration)
				In‐clinic depression severity measures (SIGHD‐IDSC)
[58]	Wearable devices with embedded sensors	Physiological and motion tracking sensors (e.g., heart rate, blood volume pulse, and skin conductance)	Monitoring physiological markers to predict depression severity and affective states (valence and arousal)	**Raw sensor data:** motion, skin conductance, heart rate, blood volume pulse
				**Signal‐to‐image transformations:** Continuous wavelet transform (CWT) and short‐time Fourier transform (STFT)
				**Affective state categories:** High/low valence and arousal
[42]	Remote measurement technologies (RMTs), including smartphones and wearables	Smartphones (passive data: GPS, Bluetooth, accelerometry) and Fitbit wearables (physical activity tracking)	Evaluate engagement with RMTs for active and passive data collection in people undergoing psychotherapy for depression	**Active**: Weekly questionnaires, speech, and cognitive tasks
				**Passive**: Smartphone sensors (GPS, Bluetooth, and accelerometry) and Fitbit data
[31]	E‐health system, combining a smart shirt and mobile application	Smart shirt (physiological monitoring) and mobile app (behavioral advice, mindfulness, and CBT)	Reduce depressive symptoms among patients with severe somatic conditions	**Passive**: Physiological data via the smart shirt
				**Active**: Behavioral and psychological inputs through the app
[52]	Portable sensor, and others	Mobile apps, neuropsychological tests, eye tracking, and EEG	Diagnostic tools for unipolar depression, assessment of behavioral and physiological biomarkers	Mood self‐assessment and cognitive tests,
				Passive behavioral monitoring for social interactions and mobility,
				Voice recording platform for vocal biomarkers,
				Neuropsychological test battery, Eye motor tracking system, EEG‐based brain activity analysis, Task for emotion perception bias quantification
[48]	Smartphone‐based application and wearable Fitbit	Smartphone app (Android/iOS) and wearable device (Fitbit)	Remote monitoring of physiological, physical, and social activities	**Smartphone apps:** collecting location, phone usage (screen status), physical activities, and sleep patterns
				**Fitbit wearable:** collecting
				activities and sleep behaviors.
[45]	Remote measurement technologies (RMTs), including smartphones and wearables	Smartphone app (Android) and wearable device (Fitbit Charge 2)	Evaluate engagement with RMTs for active and passive data collection	**Passive:** pRMT app collecting data on ambient noise, ambient light, GPS location, Bluetooth connectivity, length and duration of calls, number of text messages and emails and weather conditions, in addition to battery life.
				**Active:** aRMT app (validated questionnaires to measure depressive symptoms and self‐esteem)
				**Wearable Fitbit:** on‐going data collection of accelerometery and heart rate
[39]	GLOBEM mobile sensing platform (smartphone‐based application and wearable device)	Smartphone (not specified) and wrist (Fitbit)	To predict daily depression scores from passive sensing data using a sensor‐aware preprocessing and regression modeling approach.	**Fitbit:** sleep and steps
				**Smartphone:** Bluetooth, calls, screen time, and location
				Pre‐processed daily behavioral features across multiple time segments (morning/afternoon/evening/night, all‐day, weekday/weekend, 7‐day, and 14‐day history).

##### 3.1.1.4 Innovative and Emerging Tools

One methodological study [59] transformed raw physiological signals from wearable sensors into image‐like representations using continuous wavelet transform and short‐time Fourier transform. These signal‐to‐image representations were then used to model affective states, distinguish between high and low valence and arousal, and predict depression severity.

##### 3.1.1.5 Effectiveness and Limitations of Sensor‐Based Digital Tools

Smartphone‐based systems that model interaction patterns, mobility, and social communications often demonstrate moderate to high predictive performance for concurrent and longitudinal depressive symptoms and, in some cases, enable prediction of daily depression scores. However, at least one study reported that passive features did not substantially improve prediction beyond standardized self‐report measures [41]. Wearable‐only and hybrid smartphone–wearable platforms provide complementary physiological and behavioral markers and have been used both for risk stratification and for delivering interventions focused on CBT, activity, and sleep across diverse clinical and nonclinical populations. At the same time, the evidence base is limited by variability in reported effect sizes, short follow‐up periods, heterogeneity in outcomes and assessment methods, and recurrent challenges in user engagement with remote measurement technologies. This underscores the need for larger and methodologically harmonized trials to establish the comparative and real‐world effectiveness of these tools.

## 4 Discussion

This systematic review examined and analyzed evidence from 41 studies on sensor‐based digital tools for the detection, monitoring, and management of depressive symptoms. Among smartphones, wearable devices, and hybrid systems, we found that these tools can capture behavioral and physiological data in daily life and use them to identify depressive states, monitor changes in symptoms, and support interventions. The tools reviewed encompassed a wide spectrum from passive sensing platforms to interactive systems. Overall, the findings support the value of integrating multidimensional active and passive data streams to better characterize and address depressive symptoms.

Smartphone‐based tools relied primarily on passively acquired behavioral signals and, in many cases, combined these data with self‐report questionnaires on mood or symptoms. These behavioral data showed meaningful associations with depressive symptoms and, in some studies, supported the prediction of treatment response or early detection of depressive episodes [21, 47, 51]. Wearable devices added complementary information on physical activity, sleep, circadian rhythms, heart rate, electrodermal activity, and other physiological signals. These devices were used both to derive digital biomarkers for risk stratification and to deliver or support interventions [24, 38, 44, 55]. Hybrid systems that combined smartphone applications with wearable devices or smart garments enabled simultaneous collection of behavioral, physiological, and self‐report data and were often used to assess engagement with remote monitoring during psychotherapy, periods of bereavement, or other high‐risk contexts [22, 26, 28, 53].

Several studies went beyond conventional metrics and explored more advanced sensing and modeling strategies. These included optomyography‐based smart glasses that recorded facial muscle activity during exposure to emotional stimuli to quantify reduced facial reactivity (hyporeactivity) in depression [38], as well as systems that transformed raw physiological signals into image‐like representations using continuous wavelet transform and short‐time Fourier transform to model affective states and predict depression severity [58].

Despite these advances, such approaches are still in their early stages. Many studies relied on relatively small samples, lacked external validation, and provided limited information on how such complex models could be integrated into clinical workflows in ways that clinicians and patients can understand and trust. Long‐term user engagement, missing data, and technical challenges of multimodal integration remain key obstacles [34, 49, 55].

The NEVERMIND e‐health system, which combines a smart shirt with a mobile application, has shown promising effects in reducing depressive symptoms in patients with severe somatic conditions [31]. The STAR‐DS smartphone application, which passively collects digital biomarkers, has demonstrated potential for predicting treatment response in adolescents with depressive disorder [21]. Other interventions targeted specific high‐risk groups, such as bereaved older adults, women in the perinatal period (pregnancy and postpartum), patients with heart failure, and employees at risk of stress‐related disorders, and used promotion of activity, behavioral activation, or self‐guided CBT to reduce depressive symptoms and enhance self‐management.

Usability and acceptance of digital tools clearly vary across age groups and demographic contexts. Younger users often appear more comfortable with smartphone applications, wearables, and continuous monitoring, whereas older adults may prefer human interactions or tools embedded in familiar clinical environments [60]. Therefore, tailoring based on age, socioeconomic status, cultural norms, and physical or cognitive impairments is essential to ensure equity and effectiveness [61, 62, 63].

Emerging technologies in the detection, monitoring, and management of depression are moving toward digital phenotyping, idiographic assessments, and precision medicine [64]. Digital phenotyping aims to characterize mental health through high‐frequency, in‐the‐moment measurement of behavior and physiology in natural environments. Idiographic approaches emphasize within‐person patterns over time and, rather than relying solely on group averages, enable identification of early, personalized warning signs. Precision mental health seeks to use these data to match interventions to individual risk profiles and response patterns [64, 65].

To realize the potential of sensor‐based tools, several fundamental challenges must be addressed. First, this ecosystem is heavily dominated by wrist‐ or ring‐worn wearables and smartphones. Although they are convenient to use, they limit the range of signals that can be captured in practice and may not be ideal for all population groups or depression contexts. Alternative designs could broaden the measurement scope.

Second, many tools focus on data such as movement, sleep, and heart rate, which represent only a subset of clinically relevant depressive symptoms. Features such as changes in appetite and weight, cognitive symptoms, subjective social experiences, and qualitative aspects of mood are often underrepresented in sensor data streams [66]. Overreliance on a narrow physiological or behavioral profile risks oversimplifying the complexity of depression and neglecting important dimensions [67].

Third, many studies rely on convenience samples or existing datasets that are limited in size and demographic diversity, raising questions about how well AI models will generalize across cultures, socioeconomic groups, genders, and age ranges [66, 67, 68].

Fourth, the increasing use of deep learning and other complex modeling techniques brings both opportunities and risks. These methods are well‐suited to learning patterns from multimodal, high‐frequency data and have shown strong technical performance in detecting depressive states and predicting trajectories of symptom change [69]. However, they are often opaque, which limits their interpretability and hinders their clinical adoption. The use of explainable AI techniques and the design of models that provide clinically meaningful and understandable outputs are essential for building trust among clinicians and service users [70, 71].

Finally, continuous monitoring of physiological and behavioral markers is not without harm. Frequent feedback about mood or physiological abnormalities may increase rumination, anxiety, or a sense of being constantly surveilled for some users. False positives and misclassification by algorithms may lead to unnecessary concern or interventions. Safeguards are therefore needed to calibrate the frequency, timing, and framing of feedback and to ensure that digital tools are embedded within supportive clinical or social contexts rather than used in isolation.

## 5 Conclusions

This systematic review shows that sensor‐enabled digital tools can meaningfully capture behavioral and physiological patterns related to depressive symptoms and, in many cases, support diagnosis, monitoring, and adjunctive intervention among diverse clinical and at‐risk groups. However, the evidence base is still at an early stage: studies are often small, methodologically heterogeneous, and based on limited populations, and are accompanied by limited external validation, incomplete reporting, and underrepresentation of older adults and other underserved groups. Advanced approaches such as multimodal fusion, digital phenotyping, and deep learning offer clear technical potential, but raise important questions regarding interpretability, equity, engagement, and the potential psychological harms of continuous monitoring. Overall, these findings suggest that sensor‐based tools should at present be regarded as experimental adjuncts rather than stand‐alone solutions.

## Author Contributions

**Milad Rahimi:** conceptualization, data curation, formal analysis, investigation, methodology, writing – original draft, writing – review and editing. **Kimia Abrishamifar:** data curation, methodology, writing – original draft. **Shadi Hazhir:** data curation, methodology, writing – original draft. **Hossein Valizadeh:** data curation, methodology, writing – original draft. **Aynaz Nourani:** conceptualization, formal analysis, investigation, methodology, project administration, resources, supervision, validation, writing – original draft, writing – review and editing. **Bahlol Rahimi:** conceptualization, formal analysis, investigation, methodology, project administration, resources, supervision, validation, writing – original draft, writing – review and editing. All authors have read and approved the final version of the manuscript. Bahlol Rahimi had full access to all of the data in this study and takes complete responsibility for the integrity of the data and the accuracy of the data analysis.

## Funding

The authors received no specific funding for this work.

## Ethics Statement

This study was approved by the Ethics Committee of Urmia University of Medical Sciences (Approval ID: IR.UMSU.REC.1403.214). This research adhered to the principles of the Declaration of Helsinki for research involving human participants and data.

## Consent

Informed consent was obtained from all participants in the included studies, ensuring their understanding of the study's purpose, procedures, and implications.

## Conflicts of Interest

The authors declare no conflicts of interest.

## Transparency Statement

The lead authors, Aynaz Nourani and Bahlol Rahimi, affirm that this manuscript is an honest, accurate, and transparent account of the study being reported; that no important aspects of the study have been omitted; and that any discrepancies from the study as planned (and, if relevant, registered) have been explained.

## Supporting information

Supplementary_file.

## Data Availability

All data and materials utilized in this study are accessible to researchers upon request through an official academic email address.

## References

[1] T. Vos , S. S. Lim , C. Abbafati , et al., “Global Burden of 369 Diseases and Injuries in 204 Countries and Territories, 1990–2019: A Systematic Analysis for the Global Burden of Disease Study 2019,” Lancet 396, no. 10258 (2020): 1204–1222. 33069326 10.1016/S0140-6736(20)30925-9 PMC7567026

[2] F. Fortunato , R. Lillini , D. Martinelli , et al., “Association of Socio‐Economic Deprivation With COVID‐19 Incidence and Fatality During the First Wave of the Pandemic in Italy: Lessons Learned From a Local Register‐Based Study,” International Journal of Health Geographics 22, no. 1 (2023): 10. 37143110 10.1186/s12942-023-00332-9 PMC10157567

[3] H. Heidari and D. A. Lawrence , “Climate Stressors and Physiological Dysregulations: Mechanistic Connections to Pathologies,” International Journal of Environmental Research and Public Health 21, no. 1 (2023): 28. 38248493 10.3390/ijerph21010028 PMC10815632

[4] K. M. Scott , C. Lim , A. Al‐Hamzawi , et al., “Association of Mental Disorders With Subsequent Chronic Physical Conditions: World Mental Health Surveys From 17 Countries,” JAMA Psychiatry 73, no. 2 (2016): 150–158. 26719969 10.1001/jamapsychiatry.2015.2688 PMC5333921

[5] M. de Groot , J. H. Shubrook , W. G. Hornsby, Jr. , et al., “Program ACTIVE II: Outcomes From a Randomized, Multistate Community‐Based Depression Treatment for Rural and Urban Adults With Type 2 Diabetes,” Diabetes Care 42, no. 7 (2019): 1185–1193. 31221693 10.2337/dc18-2400 PMC6609961

[6] A. Sandmeir , D. Schoenherr , U. Altmann , C. Nikendei , H. Schauenburg , and U. Dinger , “Depression Severity Is Related to Less Gross Body Movement: A Motion Energy Analysis,” Psychopathology 54, no. 2 (2021): 106–112. 33647901 10.1159/000512959

[7] J. Chang , “Does Digital Technology Promote Ecological Civilization Construction? Evidence From China,” Environmental Science and Pollution Research 31, no. 49 (2024): 59219–59237. 39348017 10.1007/s11356-024-35156-y

[8] R. Merchant , J. Torous , E. Rodriguez‐Villa , and J. A. Naslund , “Digital Technology for Management of Severe Mental Disorders in Low‐Income and Middle‐Income Countries,” Current Opinion in Psychiatry 33, no. 5 (2020): 501–507. 32520747 10.1097/YCO.0000000000000626 PMC7398830

[9] D. C. Mohr , K. Shilton , and M. Hotopf , “Digital Phenotyping, Behavioral Sensing, or Personal Sensing: Names and Transparency in the Digital Age,” npj Digital Medicine 3 (2020): 45. 32219186 10.1038/s41746-020-0251-5 PMC7096455

[10] C. Del‐Valle‐Soto , R. A. Briseño , L. J. Valdivia , and J. A. Nolazco‐Flores , “Unveiling Wearables: Exploring the Global Landscape of Biometric Applications and Vital Signs and Behavioral Impact,” BioData Mining 17, no. 1 (2024): 15. 38863014 10.1186/s13040-024-00368-y PMC11165804

[11] V. De Angel , S. Lewis , K. White , et al., “Digital Health Tools for the Passive Monitoring of Depression: A Systematic Review of Methods,” npj Digital Medicine 5, no. 1 (2022): 3. 35017634 10.1038/s41746-021-00548-8 PMC8752685

[12] V. de Angel , S. Lewis , K. M. White , F. Matcham , and M. Hotopf , “Clinical Targets and Attitudes Toward Implementing Digital Health Tools for Remote Measurement in Treatment for Depression: Focus Groups With Patients and Clinicians,” JMIR Mental Health 9, no. 8 (2022): e38934. 35969448 10.2196/38934 PMC9425163

[13] K. Wyant , H. Moshontz , S. B. Ward , G. E. Fronk , and J. J. Curtin , “Acceptability of Personal Sensing Among People With Alcohol Use Disorder: Observational Study,” JMIR mHealth and uHealth 11 (2023): e41833. 37639300 10.2196/41833 PMC10495858

[14] R. Shaikh and S. Bhattacharya , “Polymeric Micelles in Colorectal Cancer Therapy: A Comprehensive Review of Nano‐Drug Delivery Strategies, Copolymer Types, Physicochemical Characteristics, and Therapeutic Applications,” Current Medicinal Chemistry 32, no. 20 (2024): 4033–4054. 10.2174/0109298673306752240726104241 39092735

[15] S. Fedor , R. Lewis , P. Pedrelli , D. Mischoulon , J. Curtiss , and R. W. Picard , “Wearable Technology in Clinical Practice for Depressive Disorder,” New England Journal of Medicine 389, no. 26 (2023): 2457–2466. 38157501 10.1056/NEJMra2215898

[16] M. Sheikh , M. Qassem , and P. A. Kyriacou , “Wearable, Environmental, and Smartphone‐Based Passive Sensing for Mental Health Monitoring,” Frontiers in Digital Health 3 (2021): 662811. 34713137 10.3389/fdgth.2021.662811 PMC8521964

[17] S. Akre , D. Seok , C. Douglas , et al., “Advancing Digital Sensing in Mental Health Research,” npj Digital Medicine 7, no. 1 (2024): 362. 39695319 10.1038/s41746-024-01343-x PMC11655837

[18] R. Dobson , L. L. Li , K. Garner , T. Tane , J. McCool , and R. Whittaker , “The Use of Sensors to Detect Anxiety for In‐the‐Moment Intervention: Scoping Review,” JMIR Mental Health 10, no. 1 (2023): e42611. 36729590 10.2196/42611 PMC9936367

[19] K. Watanabe , S. Okusa , M. Sato , H. Miura , M. Morimoto , and A. Tsutsumi , “mHealth Intervention to Promote Physical Activity Among Employees Using a Deep Learning Model for Passive Monitoring of Depression and Anxiety: Single‐Arm Feasibility Trial,” JMIR Formative Research 7 (2023): e51334. 37976094 10.2196/51334 PMC10692887

[20] S. Ware , C. Yue , R. Morillo , et al., “Automatic Depression Screening Using Social Interaction Data on Smartphones,” Smart Health 26 (2022): 100356.

[21] J. S. Kim , B. Wang , M. Kim , et al., “Prediction of Diagnosis and Treatment Response in Adolescents With Depression by Using a Smartphone App and Deep Learning Approaches: Usability Study,” JMIR Formative Research 7 (2023): e45991. 37223978 10.2196/45991 PMC10248781

[22] S. T. Stahl , S. F. Smagula , M. A. Dew , R. Schulz , S. M. Albert , and C. F. Reynolds 3rd , “Digital Monitoring of Sleep, Meals, and Physical Activity for Reducing Depression in Older Spousally‐Bereaved Adults: A Pilot Randomized Controlled Trial,” American Journal of Geriatric Psychiatry 28, no. 10 (2020): 1102–1106. 10.1016/j.jagp.2020.02.013 PMC7483357 32265094

[23] F. F. Sead , J. Makasana , A. Kumar Ray , et al., “Effectiveness of Mobile‐Based Digital Cognitive Behavioral Therapy for Late‐Life Depression: A Randomized Controlled Trial in Older Adults,” Cognitive Therapy and Research (2025): 1–9.

[24] H. Zhou , F. Al‐Ali , G. E. Kang , et al., “Application of Wearables to Facilitate Virtually Supervised Intradialytic Exercise for Reducing Depression Symptoms,” Sensors 20, no. 6 (2020): 1571. 32178231 10.3390/s20061571 PMC7146558

[25] K. Yokomitsu , R. Oimatsu , S. H. Y. Toh , and O. Sündermann , “Assessing the Efficacy of the INTELLECT Cognitive Behavioral Therapy Mobile App for Anxiety and Depressive Symptoms Among At‐Risk Japanese Employees: Randomized Controlled Trial,” JMIR mHealth and uHealth 13 (2025): e60871. 40554801 10.2196/60871 PMC12212887

[26] S. Jeong , C. Cha , S. Nam , and J. Song , “The Effects of Mobile Technology‐Based Support on Young Women With Depressive Symptoms: A Block Randomized Controlled Trial,” Medicine (Baltimore) 103, no. 1 (2024): e36748. 38181292 10.1097/MD.0000000000036748 PMC10766295

[27] Y. H. Lin , C. Y. Wu , B. S. Gau , C. H. Lin , H. Y. Ho , and M. F. Lou , “Effectiveness Study of a Cultural Adaptation of Cognitive‐Behavioural Therapy‐Based Application for Depressive Symptoms in College Students: A Randomised Controlled Trial,” Journal of Psychiatric and Mental Health Nursing 32, no. 3 (2025): 712–722. 39692262 10.1111/jpm.13146

[28] K. M. White , E. Carr , D. Leightley , et al., “Engagement With a Remote Symptom‐Tracking Platform Among Participants With Major Depressive Disorder: Randomized Controlled Trial,” JMIR mHealth and uHealth 12 (2024): e44214. 38241070 10.2196/44214 PMC10837755

[29] N. Rafat , F. Bakouei , M. A. Delavar , and H.‐A. Nikbakht , “Preventing Postpartum Depression in Pregnant Women Using an App‐Based Health‐Promoting Behaviors Program (Pender's Health Promotion Model): A Randomized Controlled Trial,” BMC Psychology 13, no. 1 (2025): 243. 40083038 10.1186/s40359-025-02547-w PMC11905543

[30] Z. T. Saleh , A. Aslanoğlu , W. T. Almagharbeh , et al., “Reducing Sedentary Behavior Improves Depressive Symptoms Among Patients With Heart Failure Enrolled in a Home‐Based Mobile Health App Cardiac Rehabilitation,” Journal of Nursing Scholarship 57, no. 3 (2025): 394–403. 39663212 10.1111/jnu.13039

[31] V. Carli , N. G. Petros , G. Hadlaczky , et al., “The NEVERMIND e‐Health System in the Treatment of Depressive Symptoms Among Patients With Severe Somatic Conditions: A Multicentre, Pragmatic Randomised Controlled Trial,” eClinicalMedicine 48 (2022). 10.1016/j.eclinm.2022.101423 PMC9092507 35706482

[32] S.‐J. Chen , J.‐Y. Que , N. Y. Chan , et al., “Effectiveness of App‐Based Cognitive Behavioral Therapy for Insomnia on Preventing Major Depressive Disorder in Youth With Insomnia and Subclinical Depression: A Randomized Clinical Trial,” PLoS Medicine 22, no. 1 (2025): e1004510. 39836656 10.1371/journal.pmed.1004510 PMC11750088

[33] T. A. Furukawa , A. Tajika , R. Toyomoto , et al., “Cognitive Behavioral Therapy Skills via a Smartphone App for Subthreshold Depression Among Adults in the Community: The Resilient Randomized Controlled Trial,” Nature Medicine 31 (2025): 1830–1839. 10.1038/s41591-025-03639-1 PMC12176654 40269333

[34] G. E. Hamlett , C. Schrader , C. Ferguson , et al., “Considering Comorbidities and Individual Differences in Testing a Gaming Behavioral Activation App for Perinatal Depression and Anxiety: Open Trial Pilot Intervention Study,” JMIR Formative Research 9 (2025): e59154. 39810410 10.2196/59154 PMC11750158

[35] R. Islam and S. W. Bae , “Facepsy: An Open‐Source Affective Mobile Sensing System‐Analyzing Facial Behavior and Head Gesture for Depression Detection in Naturalistic Settings,” Proceedings of the ACM on Human‐Computer Interaction 8, no. MHCI (2024): 1–32.39286336

[36] Y. Wasserzug , Y. Degani , M. Bar‐Shaked , et al., “Development and Validation of a Machine Learning‐Based Vocal Predictive Model for Major Depressive Disorder,” Journal of Affective Disorders 325 (2023): 627–632. 36586600 10.1016/j.jad.2022.12.117

[37] M. Zakariah and Y. A. Alotaibi , “Unipolar and Bipolar Depression Detection and Classification Based on Actigraphic Registration of Motor Activity Using Machine Learning and Uniform Manifold Approximation and Projection Methods,” Diagnostics 13, no. 14 (2023): 2323. 37510067 10.3390/diagnostics13142323 PMC10377958

[38] M. J. Broulidakis , I. Kiprijanovska , L. Severs , et al., “Optomyography‐Based Sensing of Facial Expression Derived Arousal and Valence in Adults With Depression,” Frontiers in Psychiatry 14 (2023): 1232433. 37614653 10.3389/fpsyt.2023.1232433 PMC10442807

[39] S. Corbett , N. Rossberg , M. M. Qirtas , and A. Visentin , eds., “Predicting Daily Depression Scores Using Passive Sensing: A Behaviour‐Aware Approach to Missing Value Handling,” in 2025 IEEE International Conference on Smart Computing (SMARTCOMP) (IEEE, 2025).

[40] F. Z. Ali , R. V. Parsey , S. Lin , J. Schwartz , and C. DeLorenzo , “Circadian Rhythm Biomarker From Wearable Device Data Is Related to Concurrent Antidepressant Treatment Response,” NPJ Digital Medicine 6, no. 1 (2023): 81. 37120493 10.1038/s41746-023-00827-6 PMC10148831

[41] A. M. Pellegrini , E. J. Huang , P. C. Staples , et al., “Estimating Longitudinal Depressive Symptoms From Smartphone Data in a Transdiagnostic Cohort,” Brain and Behavior 12, no. 2 (2022): e02077. 35076166 10.1002/brb3.2077 PMC8865149

[42] V. de Angel , F. Adeleye , Y. Zhang , et al., “The Feasibility of Implementing Remote Measurement Technologies in Psychological Treatment for Depression: Mixed Methods Study on Engagement,” JMIR Mental Health 10 (2023): e42866. 36692937 10.2196/42866 PMC9906314

[43] S. Ware , C. Yue , R. Morillo , et al., “Predicting Depressive Symptoms Using Smartphone Data,” Smart Health 15 (2020): 100093.

[44] A. Ortiz , A. Hintze , R. Burnett , et al., “Identifying Patient‐Specific Behaviors to Understand Illness Trajectories and Predict Relapses in Bipolar Disorder Using Passive Sensing and Deep Anomaly Detection: Protocol for a Contactless Cohort Study,” BMC Psychiatry 22, no. 1 (2022): 288. 35459150 10.1186/s12888-022-03923-1 PMC9026652

[45] F. Matcham , C. Barattieri di San Pietro , V. Bulgari , et al., “Remote Assessment of Disease and Relapse in Major Depressive Disorder (RADAR‐MDD): A Multi‐Centre Prospective Cohort Study Protocol,” BMC Psychiatry 19, no. 1 (2019): 72. 30777041 10.1186/s12888-019-2049-z PMC6379954

[46] G. D. Price , M. V. Heinz , A. C. Collins , and N. C. Jacobson , “Detecting Major Depressive Disorder Presence Using Passively‐Collected Wearable Movement Data in a Nationally‐Representative Sample,” Psychiatry Research 332 (2024): 115693. 38194801 10.1016/j.psychres.2023.115693 PMC10983118

[47] Y. Wang , X. Ren , X. Liu , and T. Zhu , “Examining the Correlation Between Depression and Social Behavior on Smartphones Through Usage Metadata: Empirical Study,” JMIR mHealth and uHealth 9, no. 1 (2021): e19046. 33404512 10.2196/19046 PMC7817363

[48] X. Xu , X. Liu , H. Zhang , et al., “GLOBEM: Cross‐Dataset Generalization of Longitudinal Human Behavior Modeling,” GetMobile: Mobile Computing and Communications 28, no. 2 (2024): 23–30.

[49] S. Yang , B. Gao , L. Jiang , et al., “IoT Structured Long‐Term Wearable Social Sensing for Mental Wellbeing,” IEEE Internet of Things Journal 6, no. 2 (2019): 3652–3662.

[50] M. Dominiak , K. Kaczmarek‐Majer , A. Z. Antosik‐Wójcińska , et al., “Behavioral and Self‐Reported Data Collected From Smartphones for the Assessment of Depressive and Manic Symptoms in Patients With Bipolar Disorder: Prospective Observational Study,” Journal of Medical Internet Research 24, no. 1 (2022): e28647. 34874015 10.2196/28647 PMC8811705

[51] X. Yang , J. Knights , V. Bangieva , and V. Kambhampati , “Association Between the Severity of Depressive Symptoms and Human‐Smartphone Interactions: Longitudinal Study,” JMIR Form Res 7 (2023): e42935. 36811951 10.2196/42935 PMC9996420

[52] O. Sverdlov , J. Curcic , K. Hannesdottir , et al., “A Study of Novel Exploratory Tools, Digital Technologies, and Central Nervous System Biomarkers to Characterize Unipolar Depression,” Frontiers in Psychiatry 12 (2021): 640741. 34025472 10.3389/fpsyt.2021.640741 PMC8136319

[53] A. Zhuparris , G. Maleki , L. van Londen , et al., “A Smartphone‐ and Wearable‐Based Biomarker for the Estimation of Unipolar Depression Severity,” Scientific Reports 13, no. 1 (2023): 18844. 37914808 10.1038/s41598-023-46075-2 PMC10620211

[54] Y. Rykov , T. Q. Thach , I. Bojic , G. Christopoulos , and J. Car , “Digital Biomarkers for Depression Screening With Wearable Devices: Cross‐Sectional Study With Machine Learning Modeling,” JMIR mHealth and uHealth 9, no. 10 (2021): e24872. 34694233 10.2196/24872 PMC8576601

[55] B. M. G. Rosa and G. Z. Yang , “A Flexible Wearable Device for Measurement of Cardiac, Electrodermal, and Motion Parameters in Mental Healthcare Applications,” IEEE Journal of Biomedical and Health Informatics 23, no. 6 (2019): 2276–2285. 31478880 10.1109/JBHI.2019.2938311

[56] J. Jin , B. Gao , S. Yang , B. Zhao , L. Luo , and W. L. Woo , “Attention‐Block Deep Learning Based Features Fusion in Wearable Social Sensor for Mental Wellbeing Evaluations,” IEEE Access 8 (2020): 89258–89268.

[57] T. N. K. Nguyen , N. K. Le , M. P. Tran , A. D. Tran , and N. T. Le , “Geometrical, Graphical, and Transformer‐Based Approaches for Recognizing Naturalistic Depression in Facial Behavior,” in 2025 International Conference on Activity and Behavior Computing (ABC) (IEEE, 2025).

[58] A. Ahmed , J. Ramesh , S. Ganguly , R. Aburukba , A. Sagahyroon , and F. Aloul , “Evaluating Multimodal Wearable Sensors for Quantifying Affective States and Depression With Neural Networks,” IEEE Sensors Journal 23, no. 19 (2023): 22788–22802.

[59] S. K. Pandey , H. S. Shekhawat , S. R. M. Prasanna , S. Bhasin , and R. Jasuja , “A Deep Tensor‐Based Approach for Automatic Depression Recognition From Speech Utterances,” PLoS One 17, no. 8 (2022): e0272659. 35951508 10.1371/journal.pone.0272659 PMC9371305

[60] J. M. Templeton , C. Poellabauer , and S. Schneider , “Using Wearable Devices to Mitigate Bias in Patient Reported Outcomes for Aging Populations,” International Conference on Wireless Mobile Communication and Healthcare (Springer, 2022).

[61] K. I. Taylor , H. Staunton , F. Lipsmeier , D. Nobbs , and M. Lindemann , “Outcome Measures Based on Digital Health Technology Sensor Data: Data‐ and Patient‐Centric Approaches,” npj Digital Medicine 3, no. 1 (2020): 97. 32715091 10.1038/s41746-020-0305-8 PMC7378210

[62] B. N. Renn , A. Pratap , D. C. Atkins , S. D. Mooney , and P. A. Areán , “Smartphone‐Based Passive Assessment of Mobility in Depression: Challenges and Opportunities,” Mental Health and Physical Activity 14 (2018): 136–139. 30123324 10.1016/j.mhpa.2018.04.003 PMC6095666

[63] J. Kamath , R. L. Barriera , N. Jain , E. Keisari , and B. Wang , “Digital Phenotyping in Depression Diagnostics: Integrating Psychiatric and Engineering Perspectives,” World Journal of Psychiatry 12, no. 3 (2022): 393–409. 35433319 10.5498/wjp.v12.i3.393 PMC8968499

[64] D. Zarate , V. Stavropoulos , M. Ball , and G. S. Collier , 2022. “Exploring the Digital Footprint of Depression: A PRISMA Systematic Literature Review of the Empirical Evidence,” BMC Psychiatry. 10.1186/s12888-022-04013-y PMC9214685 35733121

[65] J. F. Boswell , L. M. Anderson , and D. H. Barlow , “An Idiographic Analysis of Change Processes in the Unified Transdiagnostic Treatment of Depression,” Journal of Consulting and Clinical Psychology 82, no. 6 (2014): 1060–1071. 25045911 10.1037/a0037403

[66] W. Wang , J. Chen , Y. Hu , et al., “Integration of Artificial Intelligence and Wearable Internet of Things for Mental Health Detection,” International Journal of Cognitive Computing in Engineering 5 (2024): 307–315.

[67] A. Abd‐Alrazaq , R. AlSaad , S. Aziz , et al., “Wearable Artificial Intelligence for Anxiety and Depression: Scoping Review,” Journal of Medical Internet Research 25 (2023): e42672. 36656625 10.2196/42672 PMC9896355

[68] W. G. Monitor , The Role of Science in Mental Health. 2021.

[69] H. Li , Y. Ji , L. Xu , et al., “Using Multimodal Data Collection System as a Research Tool in the Major Depressive Disorder Analysis: A Cross‐Sectional Study Protocol,” preprint, medRxiv, 2024, 2024.07.21.24310061.

[70] C. M. Ulrich , G. Demiris , R. Kennedy , and E. Rothwell , “The Ethics of Sensor Technology Use in Clinical Research,” Nursing Outlook 68, no. 6 (2020): 720–726. 32622646 10.1016/j.outlook.2020.04.011

[71] S. Lee , H. Kim , M. J. Park , and H. J. Jeon , “Current Advances in Wearable Devices and Their Sensors in Patients With Depression,” Frontiers in Psychiatry 12 (2021): 672347. 34220580 10.3389/fpsyt.2021.672347 PMC8245757

